# Study on the eye movement characteristics of the badminton practitioners of different levels regarding visual attention

**DOI:** 10.3389/fpsyg.2022.1026006

**Published:** 2023-02-17

**Authors:** Yanlan Chen, Hutkemri Zulnaidi, Syed Kamaruzaman Bin Syed Ali

**Affiliations:** Department of Mathematics and Science Education, Faculty of Education, University of Malaya, Kuala Lumpur, Malaysia

**Keywords:** eye movements, attention choice, cognitive decision-making, intuitive decision-making, different sports levels, badminton practitioner

## Abstract

Badminton is a highly sophisticated, fierce, and competitive tactical game. It requires the same action of hitting a ball with different landing points. Therefore, the complexity of badminton practitioner's sports decision-making is relatively high. Accordingly, it is extremely important to study the difference between the eye movement characteristics of different levels of badminton athletes and the difference between the eye movement characteristics of different sports levels of amateur athletes. Overall, 15 students from the badminton professional training team of the Physical Education College of the Jiangxi Science and Technology Normal University and 15 students from the common public sports and badminton course were included as experimental participants in the present study. The laboratory experimental test on the virtual sports situation in badminton was conducted using an eye tracker. The eye movement index of both the badminton professionals and the experimental participants was recorded for statistical analysis, and the following results were obtained: (1) In the cognitive decision-masking task, the reaction time of the professional badminton practitioners was faster than that of the amateur practitioners. Similarly, in the intuitive decision-masking task, the reaction time and accuracy of the former were better than those of the latter. (2) The professional badminton practitioners' group was able to process and integrate the searched information in the process of sports attention selection information; although the amateur group was able to search and filter information, they were passive and could not actively process and assimilate the searched information. (3) The professional badminton practitioners could allocate their attention reasonably and process information in the process of attention transfer, while their amateur counterparts were affected easily by external interference factors. The level of motor intelligence of badminton practitioners in the professional group was higher than that of the amateur practitioners. Thus, these two groups of different levels showed attention transfer. (4) The mental skills of the professional group were higher than those of the amateur group.

## 1. Introduction

### 1.1. Background

Badminton is a fiercely competitive and a high-strategy open sports; however, the game also involves player's fine movements and complex and changeable technical tactics. Its offensive and defensive transitions are fast, the situation on the field is volatile, and the pace of the game is rapid and exceedingly intense. The badminton practitioners have to perform a real level of movement on the playing field; thus, based on the actual situation in the playing field, they must learn to choose the right move at the right time to use accurate techniques reasonably well to deal with the situation during the fierce competition that takes place at any time (An, 2015). Therefore, usually when playing badminton, the practitioners primarily rely on vision as well as fast and accurate search for valuable information, while disregarding the other sensory data. This approach helps to predict what is likely to happen on the playing field and react accordingly. It also determines the quality of the subsequent information processing; furthermore, the precondition for these practitioners to complete technical and tactical cooperation involves the combination of the entire tactical decision-making and technical effects.

Visual search is a classic paradigm that cognitive psychologists have used extensively to assess the assignment of attention in complex situations; it refers to the process by which individuals direct their visual attention to environmentally related cues that enable them to determine how to prepare and perform the operation in a particular situation (Pan et al., 2007). Visual search in motion is the process by which practitioners quickly search for the information they need in complex motion situations and process it into useful information (Li C., 2022). The main research of motion vision search includes time masking, event masking method, and eye movement recording, among which the former two mainly use the technical simulation of the movement technology scenario. The supposed eye movement recording method is employed chiefly to test the eye movements of subjects. Prior studies have indicated that the visual research strategies of expert practitioners are more appropriate and effective than those of their novice counterparts (Li Z. K., 2022). The visual information of every second and frame of the practitioner's movement can be obtained through the eye movement recording method, unlike other research approaches.

In this study, the eye movement recording method was used to examine the eye movement characteristics of visual attention of the badminton practitioners of different levels in a laboratory virtual movement situation. The badminton practitioners need to analyze and determine their opponent's tactical intention; only through correct judgment can they apply their own techniques and tactics more effectively, master the initiative, and win the match (Li, 2011). Thus, visual search is the first step for them to act by making the response decision. Currently, most of the research works on the visual search pattern analyze a certain technical action. However, studies that have analyzed the eye movement characteristics with regard to visual attention of such practitioners through different types of decision balls are thus far insufficient.

### 1.2. Purpose and significance

By assessing the eye movement characteristics with regard to sports attention selection of the badminton practitioners of different levels, the discrepancies in these characteristics could be explored. Combined with the subjective strategy reporting method, the differences in the eye movement characteristics and the decision-making cognitive strategies of the badminton practitioners of different levels could be revealed deeply. This approach would help in finding the most reasonable and optimal visual attention features and decision-making cognitive strategies.

The significance of this study is reflected mainly in two aspects. First, theoretically, it enriches the research on the sports decision-making cognition of badminton practitioners and brings in innovation to the eye movement theory pertaining to badminton. Second, practically, the effective conclusion of this study provides a decision-making basis for the selection of the pertinence of scientific training and the training methods for badminton practitioners; furthermore, it could improve the effect of sports training for them.

### 1.3. Research questions

1. What was the average response time and accuracy of the two groups of subjects in different decision types?
2. What were the eye movement characteristics of the two groups of subjects of the cognitive decision-making task in the visual search phase before the batting response?
3. What were the eye movement characteristics of the two groups of subjects in the intuitive decision-making task in the visual search phase before the batting response?
4. How often did subjects in both groups of the cognitive decision-making task look at different areas of interest during the batting phase?
5. How often did the two groups of subjects with the intuitive decision-making task gaze at different areas of interest during the batting phase?
6. What were the eye movement characteristics of the two groups of subjects in the cognitive decision-making task during the subsequent attention phase after hitting the ball?
7. What were the eye movement characteristics of the two groups of subjects in the intuitive decision-making task during the subsequent attention phase after hitting the ball?

## 2. Materials and methods

### 2.1. Participants

In this study, based on the expert–novice paradigm, 15 students from the badminton training team of the Jiangxi University of Science and Technology were selected as the professional subject group, among them, 7 young men and 8 young women, including 9 college students majoring in physical education and 6 master's degree students majoring in physical education; the students in the professional group had been playing sports for 5–20 years and were aged 21–35 years.; The amateur subjects group was drawn up from 15 college students of other majors who did not participate in any professional badminton training, among them, 8 young men and 87 young women, aged between 18 and 23 years, and with 0–1 year of sports experience. The average age and average years of training of the subjects in both groups are shown in Table 1.

**Table 1:** Average age and average years of training of subjects in both groups (unit: age, year).

	**M**±**SD**	
	**Average age**	**Training years**
Professional group	26.40 ± 4.614	9.07 ± 4.906
Amateur group	21.07 ± 1.486	0.320 ± 0.4039

In the experiment, two groups of subjects wore eye trackers to watch 6 randomly played badminton videos and made corresponding movements to catch the ball. The corrected visual acuity of both groups was above 1.0; moreover, there was no astigmatism. In accordance with the aim of the present research, a comparative study was conducted between the two groups. After the 30 subjects completed the test, they were interviewed. The interview comprised watching their own test video. The whole process was recorded and videotaped, thus ensuring that the interview data obtained were scientific and effective; simultaneously, they were used as a reference for the experimental results.

### 2.2. The experimental materials

#### 2.2.1. Experimental equipment

The experimental equipment and material used were the iView ETG 2.0 eye tracker and a pre-recorded badminton video, respectively. First, a badminton expert was invited to record the video consisting of six badminton technical actions: serve, back court high shot, intercept at the net, flat lob, flat stroke at the net, and smash. These badminton technical actions were obtained after the researchers interviewed the provincial badminton team coaches, some university teachers, and related scholars, and discussed them repeatedly.

#### 2.2.2. Video material

The video was recorded at 10:00 am, and the location was selected as the Badminton Stadium in the Gymnasium of Jiangxi Science and Technology Normal University. Because the venue was not a closed site, but one surrounded by the white wall, after testing, the badminton game was used to watch vaguely from the camera during the test, and it was impossible to see the flight route and falling point of the badminton ball. It was found that the entire flight route and falling point of the ball can be seen, so shooting the video is a black badminton. To ensure that the video recording is clear enough and less jittery, a tripod is fixed for recording the shooting. The position of the tripod is selected after the serve. In order not to affect the attention of the recording personnel in the experiment, the person who supplied the ball stood behind the camera and provided the ball. The person who supplies the ball cannot appear in the video, nor can the ball and volley used by the person supplying the ball appear in the video. While recording the badminton technical action video, a camera was installed behind the doubles line at the badminton net to record the entire experimental video recording process, providing reference for badminton experts to choose technical actions. Subsequently, each technique was filmed, resulting in six videos. Moreover, since each technique was repeated 10 times, there were overall 60 video clips. To ensure the accurate measurement of each subject's eye movement characteristics, the videos were sent to the badminton experts, who would select appropriate practitioners; after repeated experiment testing and an extended discussion, the good quality and the most representative videos of the six badminton techniques were chosen and compiled into a video. Additionally, the technical action in this video was played randomly.

#### 2.2.3. Experimental scene layout

**Figure 1:**
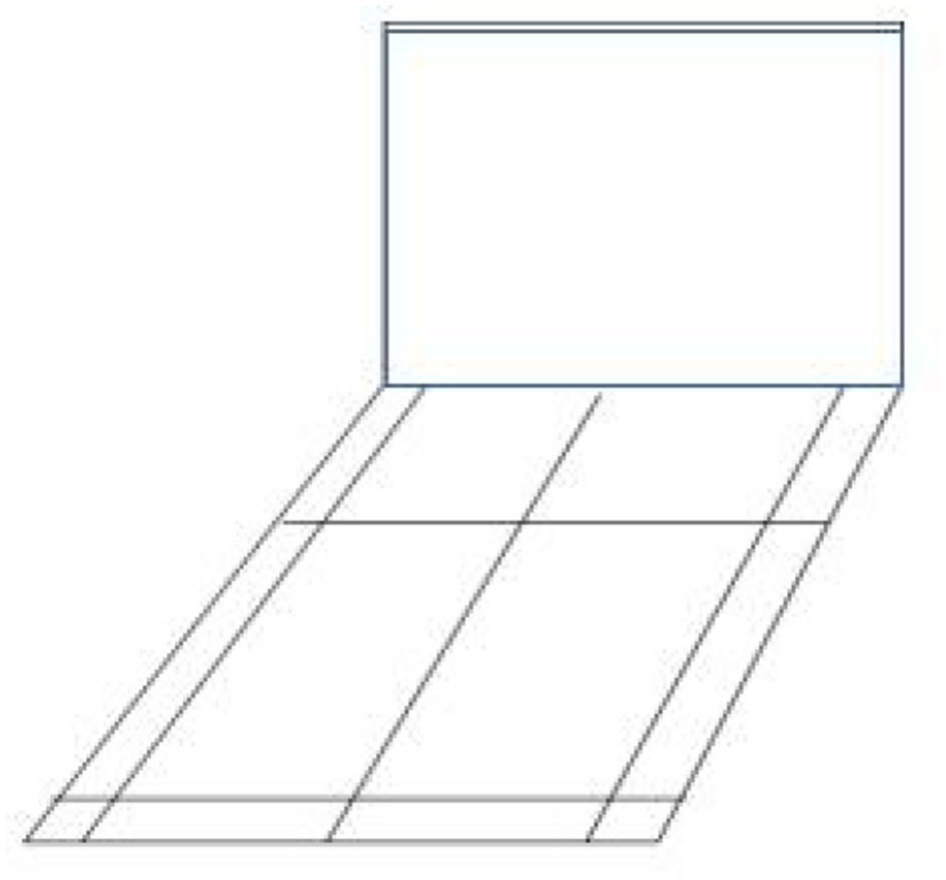
A schematic diagram of the experimental test site setting.

This experiment adopted mainly the video projection method. The projector's position was adjusted in the badminton court according to the original proportion of the figures and the placement was chosen during the video filming process; thus, the projected effect was approximately the same as the scene of a real badminton match. A badminton half court was set in front of the projection screen, and the subjects stood at the normal badminton match position. The setting diagram of the experimental test site can be seen in Figure 1.

### 2.3. Experimental design and process

The experiment used a 2 × 2 experimental design, with factors including the level of exercise (professional and amateur) and the type of exercise decision (cognitive and intuitive).

The independent variable of this study was the subjects' exercise level, while the dependent variable of this study pertained to eye movement characteristics of attention; furthermore, the test index included the area of interest of fixation, the number of fixation points, and the fixation time.

In this study, motor decision-making was divided into cognitive decision-making and intuitive decision-making; differences in the visual choice of different interest areas between the two groups in two different decision-making tasks were investigated. In the cognitive decision-making task, the video duration was 1,000 milliseconds (ms) that was set after the research of the badminton experts. In badminton, after the ball touches the racket, the ball flies to the extended surface above the net; this time was employed as the decision time of 1,000 ms.

**Figure 2:**
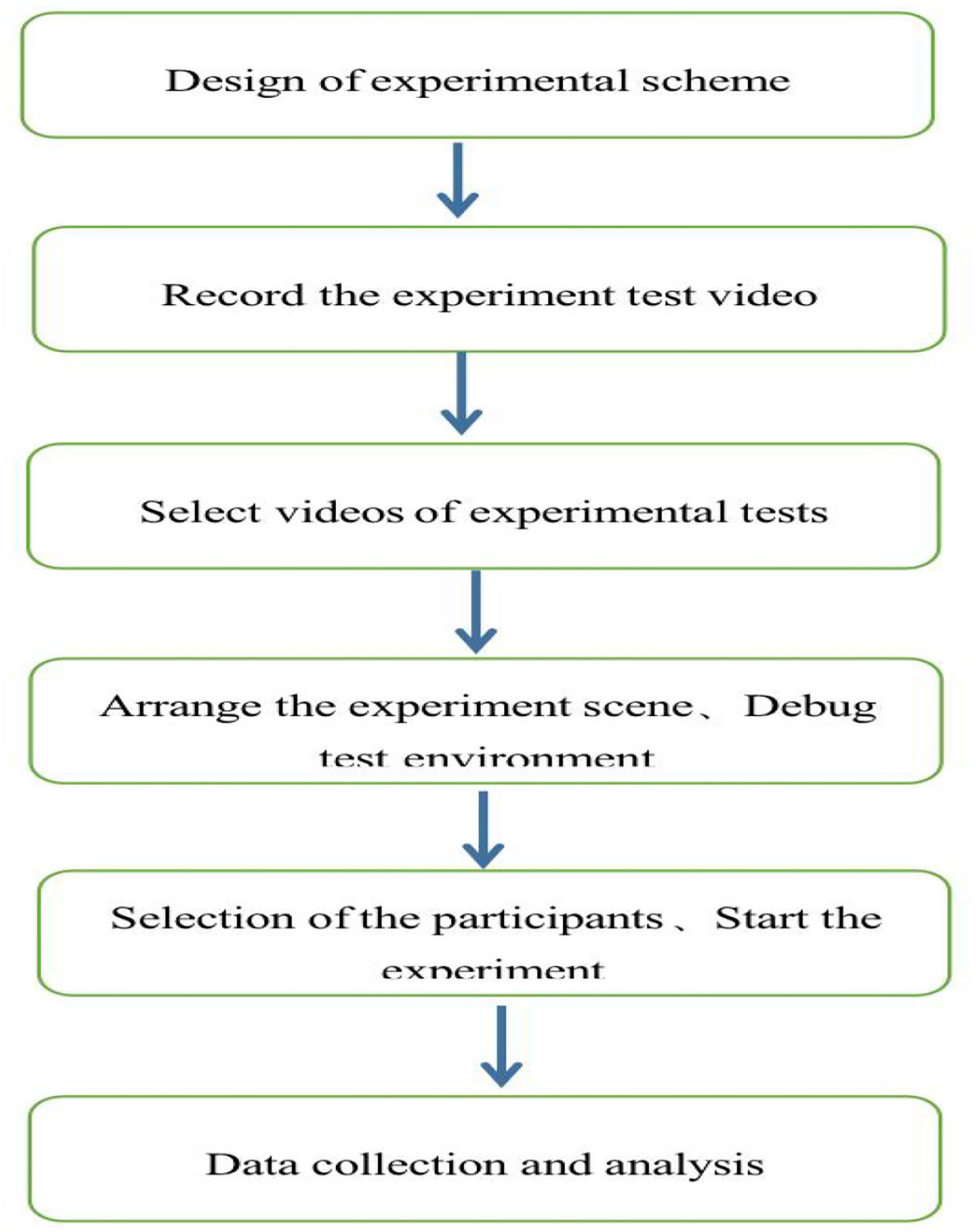
A schematic diagram of the experimental process.

Before the initiation of the experiment, the positions of both the projector and the screen were adjusted to ensure that the size of the projected image was consistent with that of an actual person to restore a real scene. The subject stood in the preparatory position with their bare hands ready to catch the ball. Subsequent to the experiment, the seriousness and importance of the investigation were explained to practitioners by the professionals; with the latter's help and guidance, the eye tracker equipment was worn. After the preparation, the experimental video was played. The six badminton technical actions recorded in the video were displayed randomly, and the subjects made the corresponding catching actions. The professional group was tested first, followed by the non-professional group. The whole process of catching by the two groups of subjects was filmed into video material for the experts to review. Finally, the total number of fixation points, the average fixation time, and the total fixation time of the two groups were obtained (The experimental flow chart is shown in Figure 2).

### 2.4. Extraction of experimental data

**Figure 3:**
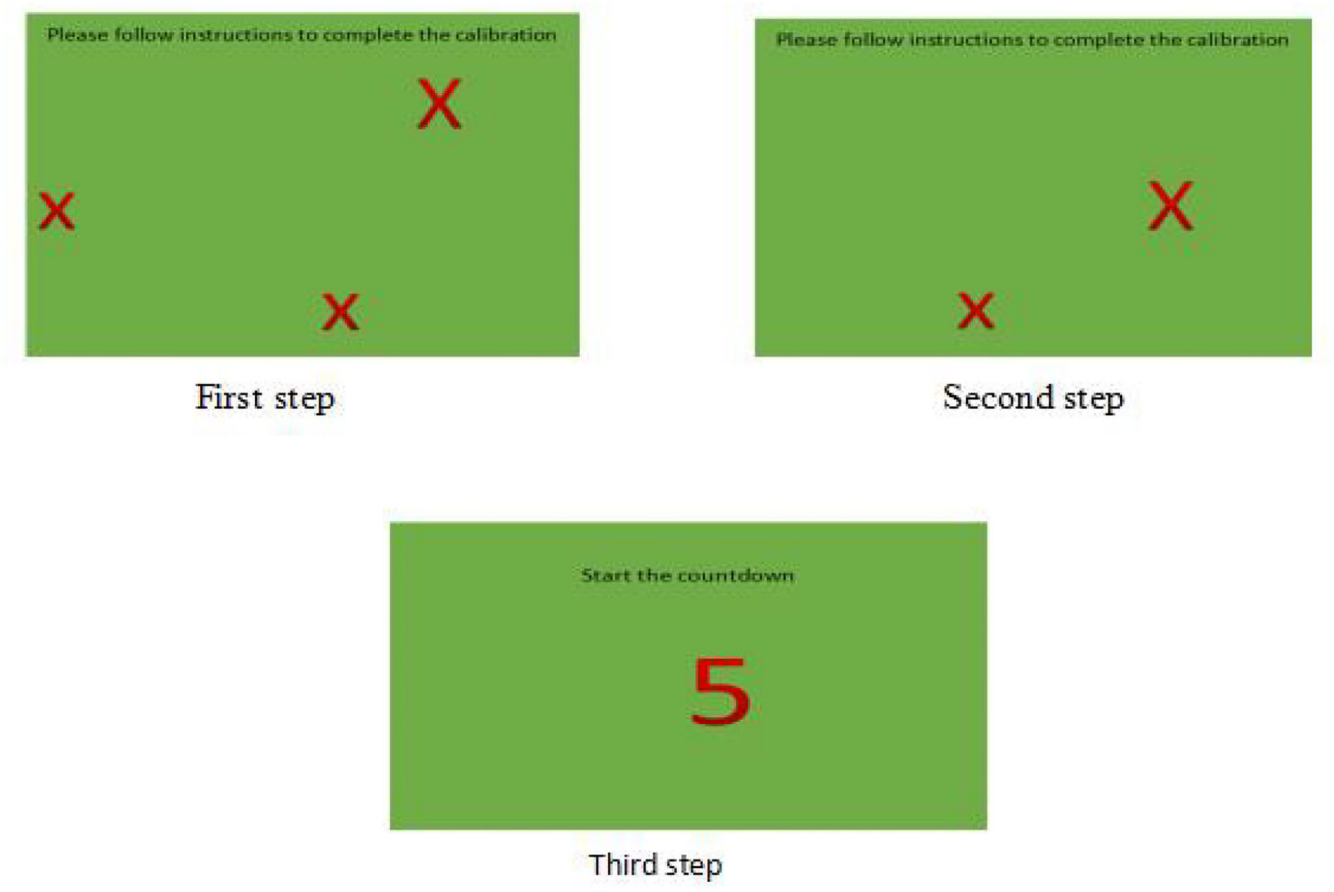
Instrument calibration procedure before the experiment.

The experiment's whole process was played using a PowerPoint Presentation (PPT). The first and second pages comprised the eye tracker calibration. If the calibration were successful, the PPT's third page would be shown and the experiment would start after a countdown of 5 s. However, if the calibration were unsuccessful, then the PPT would return to the first page, with the calibration beginning again from the three points. This is shown in Figure 3.

#### 2.4.1. Extraction method of the eye movement data

The eye movement data of the two groups of subjects were automatically generated by the eye movement recorder, and each participant had to generate an experiment and a video file. When all participants completed the task, after the experiment, they begin to use Dartfish software to play the video recording material and the experiment materials frame by frame and observe each frame of every participant with regard to changes in the fixation point of interest in different areas. The fixation time of the different areas of interest was recorded; furthermore, all data files and video recording materials were sorted and analyzed. Finally, all valid data were analyzed by the SPSS 23.0 software.

#### 2.4.2. Region of interest selection method

Using the experiment and the data analysis, the subjects were observed while executing the ball movement; the regions of their eye gaze were divided as follows: the head, the body, the arms, the balls, the rackets, the lower limbs, and *in vitro*. Among them, when the participants watched the body from the head to above the waist, it was considered as the trunk; from the shoulder to the wrist as the arm, the position where they gazed at the ball as the ball; the place where they gazed at the racket as the racket; and from below the torso of the practitioner and the pace of the practitioner's movement as the lower limbs. In addition to the above position, when the subjects gazed at a place outside the practitioner's body, it was called the outside body.

#### 2.4.3. The selection method of the gaze time stage

This article selected three experimental stages through repeated experiments and expert visits and investigations from the coach giving the ball out to the tester hitting the ball, the reaction of the receiver after the ball passed the net, the racket ball in the first two frames, called before hitting reaction visual search phase, the second phase to phase, from the racket ball before two frames to two frames, called stage to hit the ball after the ball. When the ball left the racket to the extended surface above the net, it was called the attention stage after hitting. In these three stages, the eye movement characteristics of the two groups of subjects with different types of decision-making tasks were recorded by the eye tracker.

#### 2.4.4. Statistical methods

The GeBaze 3.1 software was used to analyze and record the data during each technical movement of each student's return stroke on a Dell laptop computer with an iView ETG 2.0 eye-tracking device. The results of the BeGaze 3.1 software were combined with the supporting data to organize the results, after eliminating invalid data, enter SPSS25.0 software, and then use the t-test of two independent samples and the chi-square test to determine the statistical significance of all valid data.

## 3. Results and discussion analysis

First, descriptive statistics and homogeneity of variance test were used to test the two groups of data. The results show that the two groups of data are in normal distribution and homogeneity of variance.

### 3.1. A comparison of the mean reactivity and accuracy of the different decision types between the two groups

The two groups of subjects in different decision-making tasks had different reaction times and accuracies. The average reaction time and accuracy of the two groups in different types of decision-making tasks are shown in Tables 2 and 3, respectively.

**Table 2:** Average reaction time of the two groups of subjects in different types of decision-making tasks (unit: ms).

**M** ±**SD**				***t***	***p***
		**Professional**	**Amateur groups**		
Cognitive decision making	Serve	482 ± 86	572 ± 96	2.734	0.011^*^
	Back court high ball	267 ± 92	353 ± 103	2.412	0.022^*^
	Intercept the ball at the net	284 ± 75	402 ± 82	4.113	0.000^***^
Intuitive decision making	Flat stroke in front of the net	86 ± 23	134 ± 40	4.029	0.000^***^
	Flat lob	108 ± 31	162 ± 46	3.770	0.000^***^
	Smash shot	95 ± 27	148 ± 39	4.327	0.000^***^

* p < 0.05;

^**^p < 0.01;

*** p < 0.001.

**Table 3:** Average accuracy of the two groups of subjects in different types of decision-making tasks (unit: percentage).

**M** ±**SD**			***t***	***p***
	**Professional**	**Amateur groups**		
Cognitive decision making	2.30 ± 0.97	2.01 ± 0.85	0.871	0.391
Intuitive decision making	1.94 ± 0.92	0.71 ± 0.88	3.742	0.000^***^

^*^p < 0.05;

^**^p < 0.01;

*** p < 0.001.

According to Tables 2, 3, by comparing the average response time and accuracy of the different types of decision-making tasks, it could be concluded from the results of the research that in cognitive decision-making, no difference was observed in average accuracy between the two groups; however, a difference was noticed in the response time.

### 3.2. The eye movement characteristics of the two groups in the three different stages of the different decision-making tasks

#### 3.2.1. The eye movement characteristics of the two groups in the visual search stage before the batting response

In the cognitive decision-making task, the two groups of subjects processed the technical action of serving, and their areas of interest were mainly the head, the ball, and the racket; as regards the high shot in the back court, the subjects in both groups focused on the head, the arms, the ball, and the racket. Furthermore, when dealing with the intercept of the ball at the net, both groups concentrated on the head, the arms, the ball, and the racket. In the intuitive decision-making task, the two groups focused on the arm, the ball, and the racket when dealing with the flat lob. As regards the flat stroke in front of the net, the two groups of subjects focused on the arm, the ball, and the racket. When handling the smash shot, both groups fixated on the main areas of interest: the head, the ball, and the racket. Table 4 shows the test results pertaining to the comparison of the number of fixation points in the different areas of interest between the two groups (unit: times). Furthermore, Table 5 displays the test results pertaining to the comparison of the fixation times in the different areas of interest between the two groups (unit: milliseconds).

**Table 4:** A comparison of the number of fixation points in the different areas of interest between the two groups (unit: times).

**M** ±**SD**					**t**	***p***
		**Interest area**	**Professional**	**Amateur groups**		
Cognitive decision making	Serve	Head	0.47 ± 0.18	1.21 ± 0.16	12.277	0.000^***^
		Ball	1.48 ± 0.24	0.63 ± 0.15	11.632	0.000^***^
	Racket	1.05 ± 0.21	1.16 ± 0.27	1.246	0.223
	Back court high ball	Head	0.95 ± 0.26	1.92 ± 0.34	8.777	0.000^***^
		Arm	0.21 ± 0.03	0.26 ± 0.08	2.266	0.031^*^
		Ball	2.04 ± 0.41	2.15 ± 0.47	0.683	0.500
		Racket	1.87 ± 0.34	0.57 ± 0.11	14.089	0.000^***^
	Intercept the ball at the net	Head	0.37 ± 0.09	2.11 ± 0.57	11.678	0.000^***^
		Arm	0.23 ± 0.08	0.24 ± 0.06	0.387	0.701
		Ball	2.06 ± 0.52	0.64 ± 0.18	9.994	0.000^***^
		Racket	1.64 ± 0.22	1.41 ± 0.28	2.502	0.018^*^
Intuitive decision making	Flat lob	Arm	0.20 ± 0.16	0.62 ± 0.21	6.161	0.000^***^
		Ball	0.72 ± 0.22	0.34 ± 0.13	5.759	0.000^***^
		Racket	0.68 ± 0.17	0.63 ± 0.19	0.760	0.453
	Flat stroke in front of the net	Arm	0.57 ± 0.19	0.82 ± 0.25	3.084	0.004^**^
		Ball	0.74 ± 0.23	0.70 ± 0.21	0.497	0.622
		Racket	0.69 ± 0.20	0.48 ± 0.14	3.332	0.002^**^
	Smash shot	Head	0.75 ± 0.12	0.52 ± 0.13	5.035	0.000^***^
		Ball	0.46 ± 0.08	0.73 ± 0.24	4.134	0.000^***^
		Racket	0.37 ± 0.06	0.31 ± 0.08	2.324	0.027^*^

* p < 0.05;

** p < 0.01;

*** p < 0.001.

**Table 5:** A comparison of the fixation times in the different areas of interest between the two groups (unit: ms).

**M** ±**SD**					***t***	***p***
		**Interest area**	**Professional**	**Amateur groups**		
Cognitive decision making	Serve	Head	35 ± 7	82 ± 23	7.571	0.000^***^
		Ball	143 ± 38	107 ± 41	2.494	0.018^*^
		Racket	131 ± 33	91 ± 24	3.797	0.000^***^
	Back court high ball	Head	127 ± 35	162 ± 45	2.378	0.024^*^
		Arm	63 ± 21	83 ± 26	2.318	0.027^*^
		Ball	164 ± 46	201 ± 39	2.376	0.024^*^
		Racket	156 ± 51	74 ± 23	5.677	0.000^***^
	Intercept the ball at the net	Head	53 ± 18	167 ± 32	12.026	0.000^***^
		Arm	74 ± 25	56 ± 11	2.552	0.016^*^
		Ball	163 ± 37	95 ± 27	5.750	0.000^***^
		Racket	147 ± 25	123 ± 21	2.847	0.008^**^
Intuitive decision making	Flat lob	Arm	31 ± 10	54 ± 12	5.703	0.000^***^
		Ball	68 ± 22	41 ± 13	4.092	0.000^***^
	Racket	61 ± 18	65 ± 17	0.626	0.536
	Flat stroke in front of the net	Arm	45 ± 14	73 ± 22	4.159	0.000^***^
		Ball	84 ± 25	70 ± 21	1.661	0.107
		Racket	71 ± 19	57 ± 16	2.183	0.037^*^
	Smash shot	Head	67 ± 19	53 ± 15	2.240	0.033^*^
		Ball	52 ± 10	61 ± 13	2.125	0.042^*^
		Racket	41 ± 11	46 ± 13	1.137	0.265

* p < 0.05;

** p < 0.01;

*** p < 0.001.

Tables 4, 5 show that in the cognitive decision-making task, badminton practitioners of dissimilar levels focus on different areas of interest at varying times. In the stage subsequent to the visual search before the hitting reaction, the professional group focused more on the ball and the racket of the opponent for a relatively long time (Zhou, 2019). However, the amateur group concentrated on the body, the arms, the head, the ball, and the racket; the time of interest in these areas was also dissimilar; although the area of interest with longer attention time is also the ball and racket, compared with the professional group, the fixation time of the amateur group is still less than that of the professional group.

#### 3.2.2. A comparison of the fixation frequency of the different interest regions in the batting movement stage between the two groups

In this study, the fixation time stage selection was defined as: from the racket ball before two frames to two frames, called stage to hit the ball after the ball, due to hit the ball stage time is short, and the ball of the intuitive decision-making task in the phase of time was exceedingly short, so looking time of the two groups of different interest subjects zone cannot be calculated, and thus, the calculation of the two groups had no frequency, and a comparison of the fixation frequency of the two groups of different areas of interest subject area monitoring frequency test results between the two groups is shown in Table 6.

**Table 6:** A comparison of the fixation frequency of the two groups of different areas of interest subject area monitoring frequency test results between the two groups (unit: times).

			**Head**	**Arm**	**Ball**	**Racket**	**Lower limb**	**Outside body**
Cognitive decision making	Serve	Professional	2	2	29	27	0	0
		Amateur groups	18	21	9	9	1	2
	Back court high ball	Professional	2	3	29	25	1	0
		Amateur groups	21	19	7	6	3	4
	Intercept the ball at the net	Professional	2	2	28	28	0	0
		Amateur groups	17	21	9	10	1	2
Intuitive decision making	Flat lob	Professional	1	2	28	27	1	1
		Amateur groups	18	21	9	9	1	2
	Flat stroke in front of the net	Professional	0	1	30	28	0	1
		Amateur groups	16	17	12	11	1	3
	Smash shot	Professional	8	4	26	22	0	0
		Amateur groups	16	14	7	8	7	8

**Figure 4:**
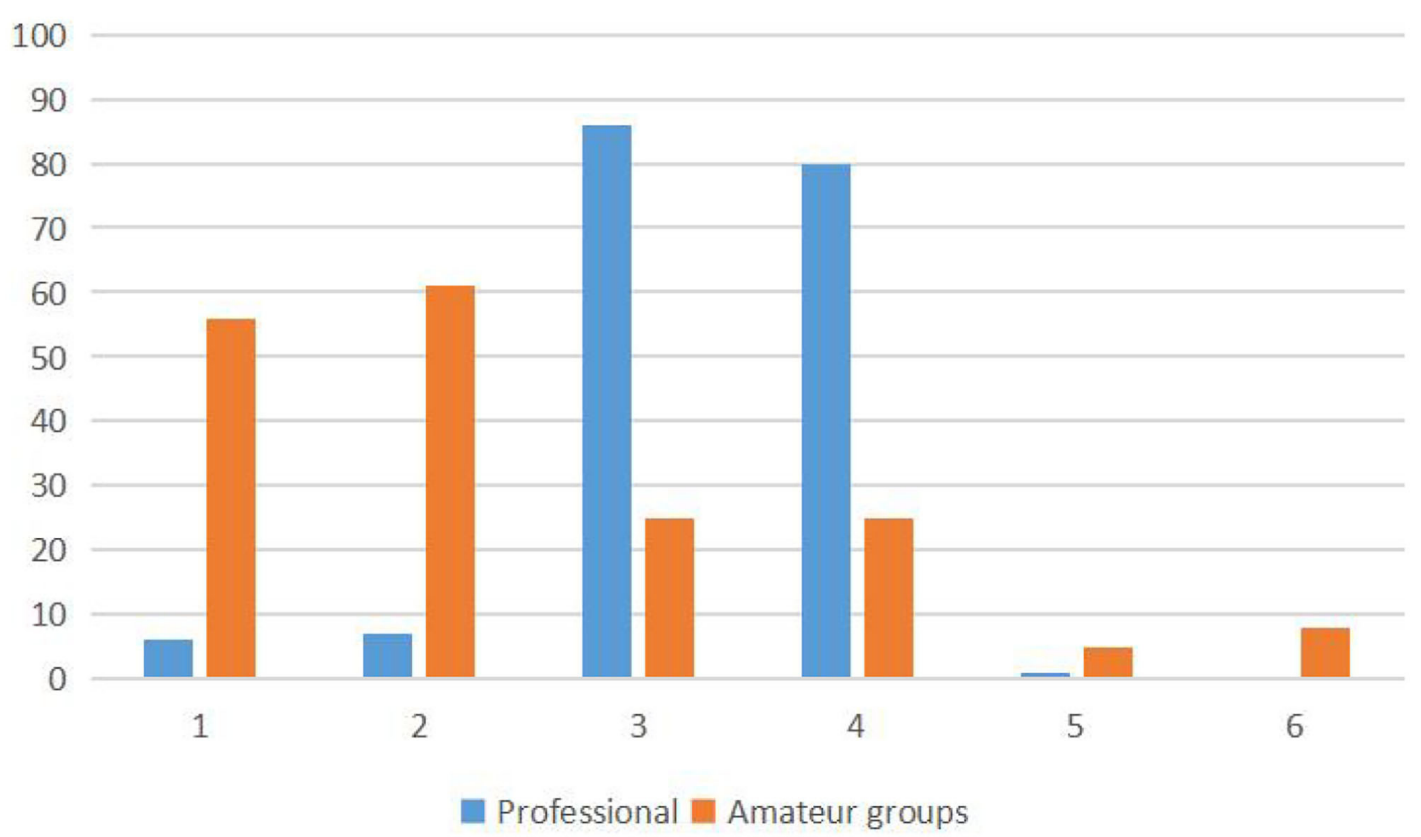
The total fixation frequency of the different regions of interest in the cognitive decision-making task between the two groups.

As observed from the data in Figure 4, differences were noticed in the total fixation frequency of the different regions of interest in the cognitive decision-masking task between the two groups. A chi-square test was conducted using A 2 × 6 contingency table, *X*^2^ = 156.204, *p* = 0.000, and the difference between the two results was very significant.

**Figure 5:**
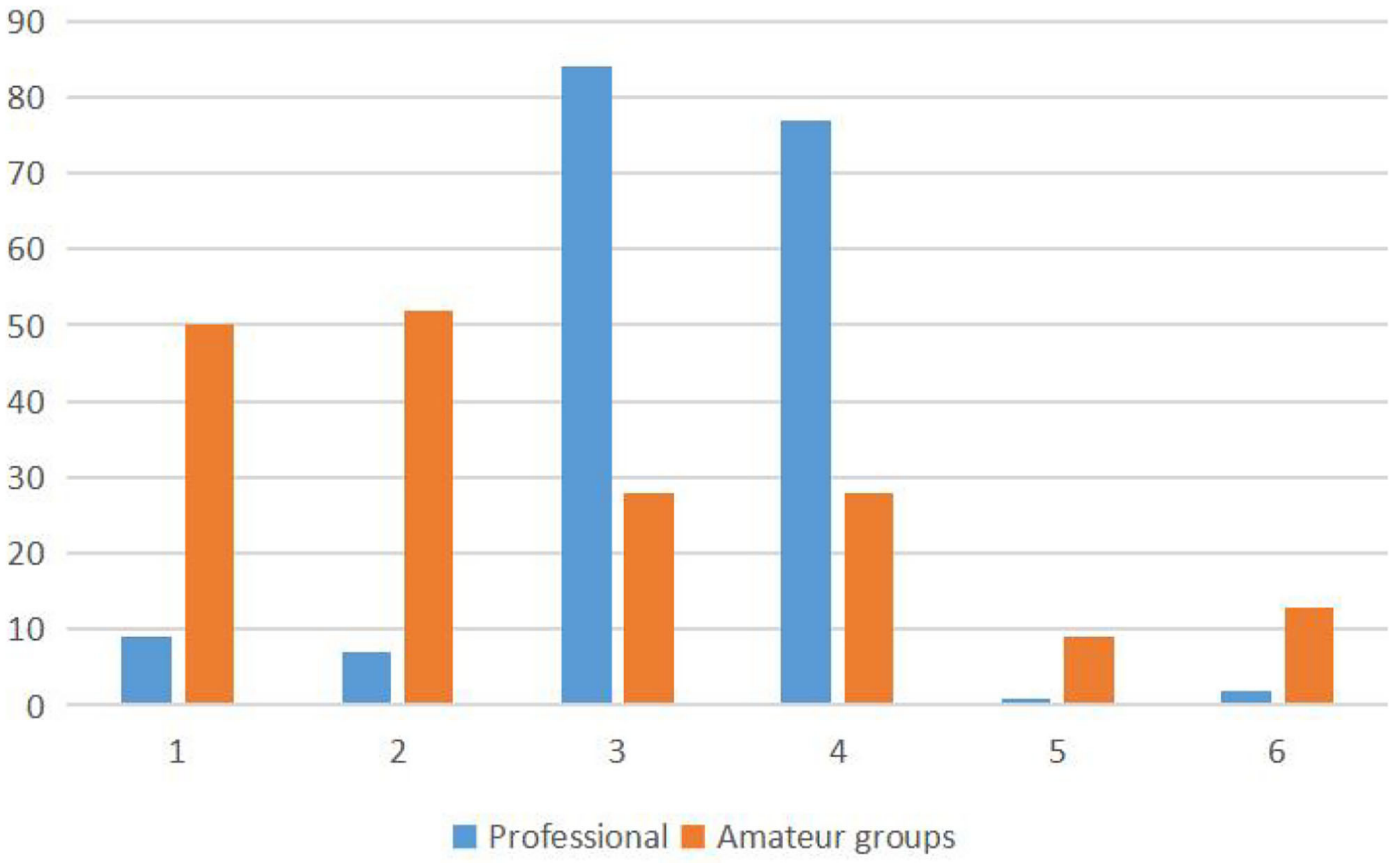
The total fixation frequency of the different interest regions in the intuitionistic decision-making task of the two groups.

As observed from the data in Figure 5, differences in the fixation frequencies of the different interest regions were observed between the two groups. A chi-square test was conducted using a 2 × 6 contingency table, X^2^ = 128.147, *p* = 0.000, and the difference between the two results was very significant.

Table 6 and Figures 4, 5 show that the fixations of the two groups were all in different interest zones.

#### 3.2.3. The eye movement characteristics of the two groups in the subsequent attention stage after hitting the ball

In the subsequent attention stage, when processing the service technical movements, the two groups focused mainly on the head, the ball, the racket, and the lower limbs; however, when processing the high ball in the back court and the cut ball in front of the net, they focused on the head, the arms, the ball, the racket, and the lower limbs. In intuitive decision-making tasks, in the two groups of participants in a shot then pay attention to the stage, deal with it clearly that they are not interested in watching the following areas: the body, the ball, and the lower limbs, with the net flat drive of the ball: the ball and the racket; and smashing the ball: the body, the ball, and the lower limbs. The test results of the comparison of the number of fixation points and times in the different areas of interest of the two groups are shown in Tables 7, 8, respectively.

**Table 7:** A comparison of the number of fixation points in the different areas of interest between the two groups (unit: times).

	**M** ±**SD**				***t***	***p***
		**Interest area**	**Professional**	**Amateur groups**		
Cognitive decision making	Serve	Head	0.31 ± 0.07	2.18 ± 0.56	12.833	0.000^***^
		Ball	2.74 ± 0.72	2.43 ± 0.65	1.238	0.226
		Racket	1.62 ± 0.38	1.67 ± 0.41	0.346	0.731
		Lower limb	1.73 ± 0.34	0.12 ± 0.03	18.269	0.000^***^
	Back court high ball	Outside body	0.65 ± 0.18	0.88 ± 0.23	3.050	0.000^***^
		Arm	0.53 ± 0.16	1.43 ± 0.34	9.276	0.000^***^
		Ball	1.07 ± 0.17	0.96 ± 0.15	1.879	0.070
		Racket	0.78 ± 0.13	0.71 ± 0.22	1.061	0.297
		Lower limb	0.94 ± 0.35	0.12 ± 0.01	9.070	0.000^***^
	Intercept the ball at the net	Head	0.74 ± 0.21	1.65 ± 0.36	8.456	0.000^***^
		Arm	1.05 ± 0.28	0.46 ± 0.13	7.402	0.000^***^
		Ball	1.27 ± 0.35	1.83 ± 0.38	4.198	0.000^***^
		Racket	0.96 ± 0.22	0.84 ± 0.24	1.427	0.164
		Lower limb	0.41 ± 0.13	0.12 ± 0.02	8.539	0.000^***^
Intuitive decision making	Flat lob	Head	0.23 ± 0.07	0.28 ± 0.08	1.822	0.079
		Ball	0.74 ± 0.11	0.82 ± 0.12	1.903	0.067
		Lower limb	0.35 ± 0.13	0.21 ± 0.02	4.122	0.000^***^
	Flat stroke in front of the net	Ball	0.61 ± 0.11	0.55 ± 0.10	1.563	0.129
		Racket	0.57 ± 0.14	0.58 ± 0.12	0.210	0.835
	Smash shot	Head	0.51 ± 0.11	0.53 ± 0.09	0.545	0.590
		Ball	0.47 ± 0.08	0.54 ± 0.13	1.776	0.086
		Lower limb	0.46 ± 0.06	0.40 ± 0.11	1.855	0.074

^*^p < 0.05;

^**^p < 0.01;

*** p < 0.001.

**Table 8:** A comparison of the fixation time in the different areas of interest between the two groups (unit: milliseconds).

**M** ±**SD**					**t**	***p***
		**Interest area**	**Professional**	**Amateur groups**		
Cognitive decision making	Serve	Head	91 ± 27	251 ± 67	8.579	0.000^***^
		Ball	308 ± 61	213 ± 54	4.516	0.000^***^
		Racket	103 ± 26	124 ± 23	2.343	0.026^*^
		Lower limb	141 ± 36	42 ± 11	10.186	0.000^***^
	Back court high shot	Head	53 ± 16	123 ± 41	6.160	0.000^***^
		Arm	47 ± 12	136 ± 44	7.558	0.000^***^
		Ball	126 ± 35	123 ± 33	0.242	0.810
		Racket	79 ± 28	104 ± 33	2.237	0.033^*^
		Lower limb	95 ± 36	34 ± 9	6.367	0.000^***^
	Intercept the ball at the net	Head	83 ± 24	175 ± 46	6.867	0.000^***^
		Arm	117 ± 27	54 ± 12	8.258	0.000^***^
		Ball	136 ± 41	210 ± 49	4.486	0.000^***^
		Racket	109 ± 21	66 ± 11	7.025	0.000^***^
		Lower limb	32 ± 14	20 ± 9	2.792	0.009^**^
Intuitive decision making	Flat lob	Head	68 ± 18	71 ± 16	0.482	0.633
		Ball	68 ± 15	67 ± 16	0.177	0.861
		Lower limb	65 ± 14	62 ± 12	0.630	0.533
	Flat stroke in front of the net	Ball	78 ± 21	76 ± 18	0.280	0.781
		Racket	74 ± 15	79 ± 22	0.727	0.473
	Smash shot	Head	59 ± 15	74 ± 17	2.562	0.016^*^
		Ball	72 ± 18	66 ± 13	1.047	0.304
		Lower limb	69 ± 13	60 ± 10	2.125	0.042^*^

* p < 0.05;

** p < 0.01;

*** p < 0.001.

From Tables 7, 8, it can be concluded that for the two groups regarding the choice of the area of interest, the visual search phase increased at a lower interest area, only in the lower limbs; the professional group's fixation point number and time was exceedingly higher than that of the amateur group.

## 4. Discussion

In the cognitive decision-making in the process of flight, the two groups had sufficient time to make a decision. Professional badminton practitioners have an extended sports life, exercise experience, and a badminton technical capacity higher than those of amateur photographers (Xu, 2017); hence, the handling of the ball, quick response and back to the ball, and the time required were lower than those of the amateur group. The average reaction time of the professional group was lower than that of the amateur group. In the intuitive decision-making task, the average reaction time and accuracy of the two groups were different. In addition, the average reaction time and accuracy of the professional group were lower and higher than those of the amateur group, respectively. In the case of more ball speed and less time to judge, the decision ability of the former was better than that of the latter.

In the process of attention selection, the professional group automatically filtered the unimportant information from their own perspective and selected the important information they needed (Sun, 2018). As early as 1958, the British psychologist Broadbent proposed the filter theory and pointed out that there was a limit to how much information our brain's nervous system can process. When the information enters our nervous system through different senses, some of it is allowed to pass through and subsequently undergo further processing due to filtering mechanisms in certain parts of the nervous system; the rest of the information is blocked out of the mechanism and is eventually lost.

In the intuitive decision-making task, the participants were required to make decisions quickly due to the extremely limited time of the game picture; thus, they were unable to analyze in a timely manner and could make decisions only by feeling. In this study, the two groups showed significant differences in the attention selection of the different interest areas during the visual search stage before hitting the ball. According to a British psychologist's filter theory, the two groups automatically filtered the two zones of interest—the lower limbs and the outside of the body—and focused on the opponent's arms, ball, and racket. They demonstrated a stable state in the fixation time of the different interest areas. The factors affecting the attention stability were mainly the intensity and duration of the stimulus and its uncertainty in time and space.

The attention was easy to shift. In the cognitive decision-making task, the professional group was able to search for their desired interest areas, and subsequently constantly change the gaze order between them, process and integrate the searched information, and finally make a response to return the ball. In the process of attention transfer, the amateur group was easily disturbed by the irrelevant external stimuli, and their attention left the interest area's range, referred to as a distraction. Differences in the ability of attention of the badminton practitioners of different levels were observed. The professional group had stronger attention ability, while that of the amateur group was weaker. In the experimental test phase of this study, a few amateurs did not pay attention to the area of interest which may be due to the influence of some emotional factors or excessive eye fatigue or tension. The reason was that when a badminton practitioner is nervous, he cannot concentrate on gazing at a certain object, because when he does so, regardless of how long the gaze continues, he would experience psychological fatigue, thus reducing his alertness (Xie and Yu, 2014). Therefore, amateur badminton practitioners tell themselves to focus more when they are more nervous; this mental effort often leads to the concentration of attention away from us.

The number of attention shifts in the two groups of different levels of badminton practitioners is relatively small, because before someone with pure tone and light spot has been tested after the experiment, paid attention to the transfer time of 40–60 ms. According to the calculation, regarding the two groups, in the intuitive decision-making task, the attention transfer occurred at most one time. Thus, in the intuitive decision-making task, the two groups made decisions more according to their own sports experience and intuition, while the amateur group did so more by conditioned reflex and intuition. Therefore, the attention transfer occurred in both the groups. The professional group could reasonably allocate attention and process information in the process of attention transfer, while the amateur group was affected easily by the external interfering factors, resulting in distraction.

The professional group could search the ball's flight path quickly and efficiently, and subsequently, starts looking at the other player's lower limbs, adjusts the stance by watching the movement of the other player's lower limbs, and prepares to catch the next ball. The main processing theory of attention was proposed as early as 1963 and then perfected by Lohrmann in 1968. This theory emphasizes mainly the full analysis of perception, response selection, and the role of memory in information selection. Therefore, it is also called the perfect processing theory, the response selection, or the memory selection theory. In a study on the eye movement characteristics and the reaction time of table tennis practitioners' expected judgment of attack line, Duan (2008) proposed that obvious differences in the visual features such as the fixation point and time between the expert group and the novice group when watching the attack line were noticed. The former was focused more on the positional information directly related to the attacking action. They paid more attention to the opponent's chest, shoulder, wrist, and racket areas. Moreover, they could search for more effective visual information than their novice counterparts.

In a study on the eye movement characteristics of tennis practitioners at different levels in the process of predicting expected drop points, He (2015) concluded that professionals and beginners display great differences in their visual search when watching tennis matches; additionally, the acquisition of information and processing of the former are more efficient than those of the latter. This aspect was reflected in the fact that the professional group focused for less time than the beginners. The type of sport examined by the aforementioned two scholars belongs to the same group of sports as that assessed in this study. Table tennis, tennis, and badminton are all net-to-net games; moreover, the conclusions of the aforementioned two scholars are consistent with those of this research. The professional group had the longest fixation time on the ball, followed by the lower limbs (Zhou, 2019); however, the amateur group basically maintained it at an average level for the different interest areas, and there was a negligible difference between the longest and the shortest time. Due to the limited time and no training, the latter had no time to react, and the opponent's batting action had ended.

Wang (2002) conducted an experimental research on intuitive decision-making tasks in handball sports which are of a different nature; thus, there are differences in the individual performance of these two kinds of tasks. In this study, the badminton practitioners in the professional group usually processed the searched information and subsequently made the corresponding decisions. The two groups of badminton practitioners at different levels will filter out some unimportant points of interest for the choice of information. However, the professional group can process and combine the information searched (Wang, 2015), but the amateur group does not have the ability to search for information actively or the ability to process information.

## 5. Conclusions and future recommendations

### 5.1. Conclusion

In the cognitive decision-making task, no significant difference was observed in the accuracy of judgments between badminton practitioners of different levels. However, a significant difference was noticed in the average reaction time, with the average reaction time of badminton practitioners in the professional group being faster than that of the amateur group. In the intuitive decision-making task, a significant difference was observed in the judgment accuracy and mean reaction time among badminton practitioners of different levels, and the judgment accuracy of the professional group was higher than that of the amateur group, and the mean reaction time was faster than that of the amateur group.

In the information processing of attention selection, the two groups of badminton players with different skill levels filtered some unimportant regions of interest autonomously. Whereas the professional group was able to process and integrate the information searched, the amateur group was passive, able to search, and filter information, but did not have the ability to actively process and integrate the information searched.

The attention-shifting process occurred in both groups of badminton practitioners at different levels. The professional group was able to allocate attention and process information reasonably well during the attention-shifting process, while the amateur group was susceptible to external distractions, which resulted in distraction.

The motor intelligence level of professional badminton practitioners was higher than that of amateur badminton practitioners. As regards casual attention, professional badminton practitioners were able to pay attention steadily and intensively, while amateur badminton practitioners found it more difficult to pay attention.

Badminton is a competitive event with complex and changeable techniques and tactics. Badminton practitioners' psychological ability and technical and tactical ability have a very close relationship, and the professional group badminton practitioners' psychological quality and psychological skills are higher than those of the amateur group.

### 5.2. Future recommendations

The eye tracker is a scientific research equipment that is specially designed in the background of the development of modern science and technology and has a relatively better value and role in the field of sports research. This study assessed the characteristics of attention selection of the badminton practitioners of different sports levels using an eye tracker, in an attempt to reveal the differences in their attention selection methods. However, a few shortcomings were observed in the experiment. First, a relatively limited number of professional practitioners took part in the experiment, among whom only a few had won the title of athlete grade. Therefore, one cannot complete the study on the characteristics of attention selection of exercisers with different exercise levels. Second, as it is in the experiment process, the video rendering time was shorter; furthermore, the real movement situation had shown a certain gap as the badminton practitioners chose continuity in the whole movement process, while the images pertaining to the design of the experiment process appeared relatively abruptly. This would affect the movement's judgment, thus influencing the experimental results to a certain extent.

Scientific evidence to reveal the rules of movement is the responsibility of contemporary sports workers. In the future research process, the experiment design and the attention characteristics of badminton practitioners should be formulated more scientifically. Such a development would help to improve the athletic level and continue to maintain its leading position in badminton worldwide.

## Data availability statement

The original contributions presented in the study are included in the article/supplementary material, further inquiries can be directed to the corresponding author.

## Ethics statement

Written informed consent was obtained from the individual(s) for the publication of any potentially identifiable images or data included in this article.

## Author contributions

YC completed the first draft of the article as well as experiments, data collection, and analysis of data. HZ and SS commented, revised, and edited the first draft of the article. All authors contributed to the article and approved the submitted version.
